# Estimating the Starling equation parameters in living humans by using volume kinetic analysis

**DOI:** 10.1007/s00424-026-03219-3

**Published:** 2026-10-12

**Authors:** Robert G. Hahn

**Affiliations:** Karolinska institutet at Danderyd sjukhus (KIDS), Stockholm, 171 77 Sweden

**Keywords:** Albumin, Capillary filtration, Crystalloid fluid, Hemodilution, Pharmacokinetics, Starling equation, Urine output

## Abstract

The transcapillary filtration of fluid is governed by the Starling forces. The purpose of the present study was to simultaneously estimate all Starling parameters in living humans by using likelihood mathematics. First, the capillary filtration was estimated by volume kinetic analysis at 3,015 time points during 193 intravenous infusion experiments using crystalloid and colloid fluid in adult volunteers and surgical patients placed in the supine position. Second, the obtained capillary filtration and the measured plasma colloid osmotic pressure were entered into a new kinetic analysis to estimate the five remaining parameters in the Starling equation. The result shows that the capillary hydrostatic pressure was 16 mmHg (P_c_), the interstitial hydrostatic pressure − 2.2 mmHg (P_i_), the interstitial colloid osmotic pressure 1.6 mmHg (π_i_), the reflection coefficient 0.79, and the capillary filtration coefficient (K_f_) 306 mL/min/mmHg for the whole body when crystalloid volume loading was performed. K_f_ was significantly lower when colloid fluid (albumin 5% and 20%) was infused, and π_i_ was lower in patients with subacute inflammation. Association plots showed that P_c_ was low in hypovolemia and inflammation but markedly higher when albumin was infused. Plots over time suggested that K_f_ was high early during an infusion, which was probably due to release of nitric oxide due to shear stress, but then decreased gradually. In conclusion, the parameters in the Starling equation could be estimated with acceptable confidence during fluid infusion experiments in humans. Changes of K_f_ appeared to be key parameter that modulated the capillary filtration rate during volume loading.

**Supplementary Information:**

The online version contains supplementary material available at https://doi.org/10.1007/s00424-026-03219-3.

## Introduction

The Starling equation is a key to understanding the forces that regulate the transcapillary leakage fluid (net fluid movement, J_v_). The equation has six parameters. These are the capillary hydrostatic pressure (P_c_), the interstitial hydrostatic pressure (P_i_), the colloid osmotic pressure of the capillaries (π_c_) and of the interstitial space (π_i_). There is also a parameter, the reflection coefficient (*σ*), that quantifies the permeability of the capillary wall to proteins, and the capillary filtration coefficient (K_f_). which is a proportionality factor for the relationship between the pressure gradients and J_v_. The Starling equation is usually written on this form:

$$J_v=K_f\left[\left(P_c-P_i\right)-\sigma\left(\pi_c-\pi_i\right)\right]$$

The parameters in the Starling equation have usually been assessed in small animals under highly controlled laboratory conditions. The works by Guyton et al. in the 1960s showed that P_i_ should be slightly negative (−2 to −3 mmHg) [1, 2] although it becomes lower in inflammatory conditions [3]. P_c_ is usually referred to as being in the range of 20–35 mmHg. The easiest parameter to access is π_c_ because it is believed to correlate closely with the colloid osmotic pressure of the plasma. Historically, π_i_ has been assumed to be governed by the protein concentration of the interstitial space, which is 40%−50% of the plasma concentration [4, 5]. However, this view has been challenged by the Revised Starling principle, which holds that π_c_ equilibrates against a thin “essentially protein-free” compartment just outside the endothelium [6, 7].

So far, there is no study that estimate all parameters in the Starling equation at the same time in living humans, but recent development in volume kinetic analysis could make this possible [8, 9]. The background is that J_v_ can be estimated during an intravenous (i.v.) bolus infusion of fluid based on repeated measurements of the urine output and the blood hemoglobin (Hb) concentration. J_v_ for the whole body is then obtained at each point in time during the experiment. If these data are combined with measurements of π_c_ we have two parameters in the Starling equation and may be able to estimate the others. Hence, the hypothesis of the present study was that estimating the Starling equation parameters is possible using this approach.

## Materials and methods

### Methodological overview

The analysis of the Starling parameters was based on mathematical comparisons between the plasma oncotic pressure (π_c_) and the calculated capillary filtration rate (J_v_) during and after 193 intravenous infusions of fluids that contained or didn´t contain albumin. J_v_ was first obtained by volume kinetic analysis based on peripheral blood sampling and measurements of urine flow [8]. The subjects, procedures, and the methodology used for this step of the analysis are described below, and results have been summarized elsewhere [9, 10]. Importantly, the pharmacokinetic model used to estimate J_v_ needs to be different depending on whether the infusion fluid is hypo- or hyper-oncotic. Therefore, the measured π_c_ and the J_v_ derived by the appropriate pharmacokinetic model were used as input data in the present second part of the analysis. These data are shown in Supplementary File 1.xls. The rationale was the same in the first and the second part of the analysis, i.e., using likelihood mathematics to estimate several unknown parameters in a differential equation where two variables were known.

### Subjects

The material for the study was derived from a database, owned by the author, that contains approximately 500 intravenous (i.v.) infusion experiments conducted in humans where the initial purpose had always been to study fluid volume kinetics. The selection criterion was that the experiment must contain serial measurements of both the blood Hb and plasma albumin concentrations during and after volume loading while no administration of catecholamines drugs was allowed. Exclusion criteria were age < 18 years, poor health status (ASA Classes III–IV), severe allergies, impaired kidney function, and heart failure. All experiments were performed in accordance with the tenets of the Declaration of Helsinki, and each subject provided written informed consent to participate before any experiment was initiated.

The selection ended up with 193 subjects from 10 studies where most of the volume kinetic data already had been published in two major reports [9, 10]. The ethics approvals and publication details of these 10 studies are given in Table S1 and the demographic details in Table 1.

**Table 1:** Demographics details about the study groups

Group	Infused fluid	Condition	Age (years)	Body weight (kg)	Mean arterial pressure (mmHg)	π_c_ (mmHg)	Infu- sions (*N*)	Data points (*N*)
0	Ringer	Healthy	30 ± 5	78 ± 10	90 ± 11	24.9 ± 1.7	30	310
1	Albumin 5%	Healthy	29 ± 8	77 ± 10	84 ± 9	25.4 ± 1.1	26	619
2	Albumin 20%	Healthy	31 ± 14	74 ± 11	91 ± 8	25.4 ± 1.1	44	548
3	Albumin 20%	Inflammation	55 ± 17	84 ± 19	79 ± 10	19.0 ± 1.6	30	383
4	Ringer	Surgery	53 ± 16	69 ± 10	70 ± 14	24.4 ± 1.2	29	568
5	Albumin 20%	Surgery	46 ± 15	70 ± 15	66 ± 8	23.9 ± 0.8	15	186
6	Ringer	Hemorrhage	28 ± 3	74 ± 7	84 ± 10	24.2 ± 0.9	19	401

The selected subjects had received either 25 mL/kg of Ringer solution (≈ 1.9 L), 10 mL/kg of albumin 5% (≈ 800 mL), or 3 mL/kg of albumin 20% (≈ 200 mL) at a constant rate over 30 min.

### Procedures

Volunteers were allowed to ingest one glass (250 mL) of liquid on the morning of the experiment, which started between 8 and 9 a.m. Surgical patients could ingest liquid for up to 2 h before the operation. All experiments were performed with the subject lying in the flat supine position on a bed and with their arms resting alongside with the torso. Measurements of the blood Hb and plasma albumin concentrations were made on venous blood obtained from a cannula inserted into the cubital vein. Blood was sampled on 15–30 occasions over 2–6 h. Specifically, blood was sampled every 5 min during each infusion and for 30 min after infusion. Thereafter, the interval was increased to 10–30 min. The excreted urine volume was measured 2–3 times per experiment. Hb and Alb were measured in the hospital’s clinical chemistry laboratory with, a given by duplicate samples taken at baseline, coefficients of variation of approximately 1% and 3%, respectively. An Osmomat 050 (Gonotec, Berlin. Germany) was used to measure the plasma colloid osmotic pressure (COP) with a coefficient of variation of 2%.

### Study groups

Infusion with Ringer´s acetate solution was given to 30 volunteers, to 29 patients undergoing surgery under general anesthesia, and to 10 volunteers who had 450 mL of blood withdrawn just before one Ringer infusion and 900 mL withdrawn just before another infusion. Albumin 5% was given to 26 healthy volunteers. Albumin 20% was administered to 44 healthy volunteers and to 15 patients undergoing minimal but lengthy (> 5 h) surgery. Albumin 20% was also infused in 30 patients with subacute inflammation. The first 15 of them were studied in the first postoperative morning after having undergone major open abdominal surgery for cancer, and the other group of 15 patients were assessed 4–7 days after burn injury that had necessitated hospital care. Inflammation was evidenced by plasma concentrations of C-reactive protein to 60–90 mg/L (normal value < 5 mg/L) (Table 1).

### Calculations

Volume kinetics is pharmacokinetics for infusion fluids. Serial measurements of hemodilution are handled to distinguish between capillary filtration (J_v_), lymphatic return, and urine output. The sizes of the body fluid spaces that become expanded by the infused fluid are also estimated [9, 10]. Technically, J_v_ is obtained as the product of the size of the central fluid space at baseline (i.e., the plasma volume), the rate constant for capillary leakage (*k*_12_), and the Hb-derived plasma dilution.

The current analysis used J_v_ and plasma albumin to estimate the other five parameters in the Starling equation (K_f_, P_c_, P_i_, *σ*, and π_i_). Technically, the derivative of J_v_ (hence, dJ_v_/dt) was entered. together with a conversion of plasma albumin to π_c_, into the Starling equation written on the form shown in the Introduction. The estimation of J_v_ (Part 1) and the subsequent analysis of the Starling parameters (Part 2) were both made using population (mixed models) kinetics as implemented in Phoenix NLME, version 8.7; Pharsight, St. Louis, MO). The sensitivity of the parameters for perturbation was tested using the “Profile” function in Phoenix.

Demographic data showing a normal distribution were reported as the mean ± standard deviation (SD). The Phoenix program used log likelihood mathematics to obtain the “best estimate” and the 95% confidence interval of each parameter in the Starling equation. A covariate was added if the 2 x log likelihood (LL) of the model decreased by > 3.8 points (*P* < 0.05).

## Results

### Population

**Fig. 1:**
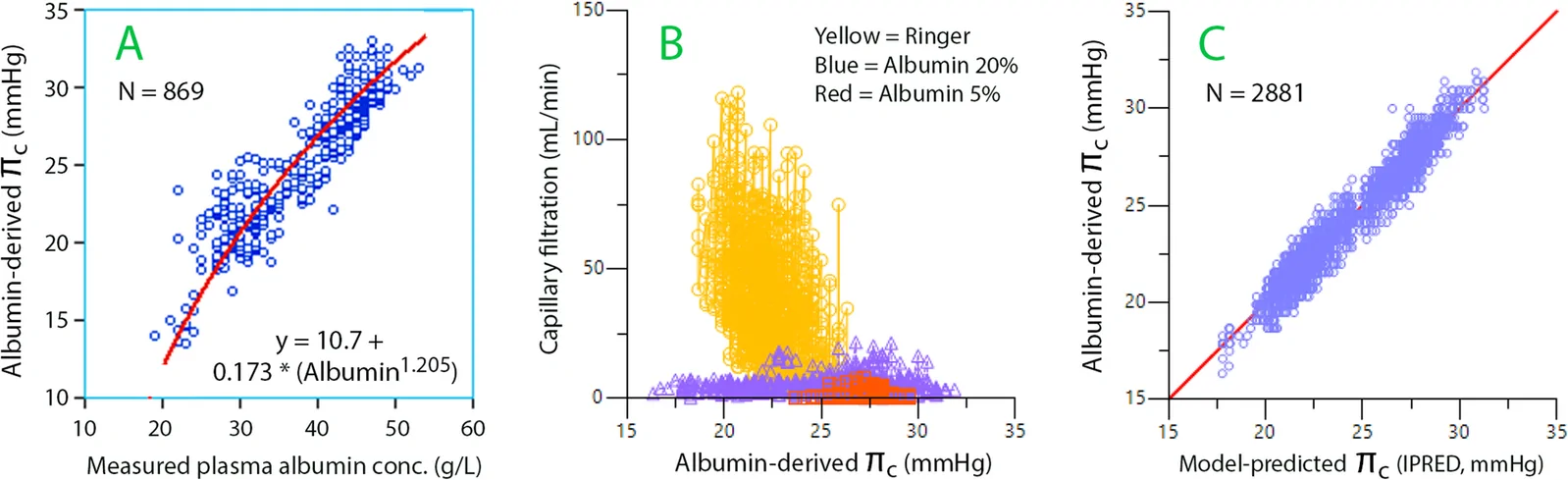
(**A**) The curvi-linear relationship between the plasma albumin concentration and the derived plasma colloid osmotic pressure (π_c_) estimated from actual measurements of this pressure (**B**) Relationship between the two variables used for estimating the parameters in the Starling equation (**C**) Agreement between the measured π_c_ and the model-predicted value in the final kinetic analysis

The analysis was based on 3,015 data points collected during 193 infusion experiments (Table 1) published in 10 studies using the same method (Table S1). The conversion of plasma albumin to plasma COP (π_c_) and the distribution of this variable between the three study groups given 0%, 5%, or 20% albumin are shown in Fig. 1A and B.

### Kinetic analysis

The final parameter estimates of the kinetic analysis are shown in in Table 2. The correlation between the albumin-derived π_c_ (dependent variable) and the values predicted by the kinetic model is illustrated in Fig. 1C. The five remaining parameters in the Starling equation were estimated with good precision, and two covariates were added. First, K_f_ obtained a lower value when albumin solution was administered instead of Ringer´s. The shown value of 306 mL/min/mmHg for K_f_ in Table 2 denotes only the Ringer experiments. The other statistically significant covariate consisted of a reducing effect of inflammation on π_i_.

**Table 2:** Parameter estimates in the Starling equation based on curve-fitting of the hemodilution and urine output during 193 volume loading experiments with albumin 5%, albumin 20%, and Ringer´s acetate. A covariate value Y changes the parameter X by X*e^Y^ where e = 2.718

	Covariate	Best estimate	95% CI	CV%	–2(LL)
*P*_*c*_ (mmHg)		15.5	12.9–18.3	8.8	
*P*_i_ (mmHg)		−2.22	−2.44 to −2.01	−4.9	
π_i_ (mmHg)		1.55	1.53–1.57	0.6	
*K*_f_ (mL/min/mmHg)		306	301–311	0.8	
*σ* reflection coefficient		0.79	0.72–0.86	4.6	7745
*K*_f_	Albumin	−0.41	−0.42 to −0.39	−2.1	7738
π_i_	Inflammation	−0.31	−22.7 to + 22.7	3661	7728

**Fig. 2:**
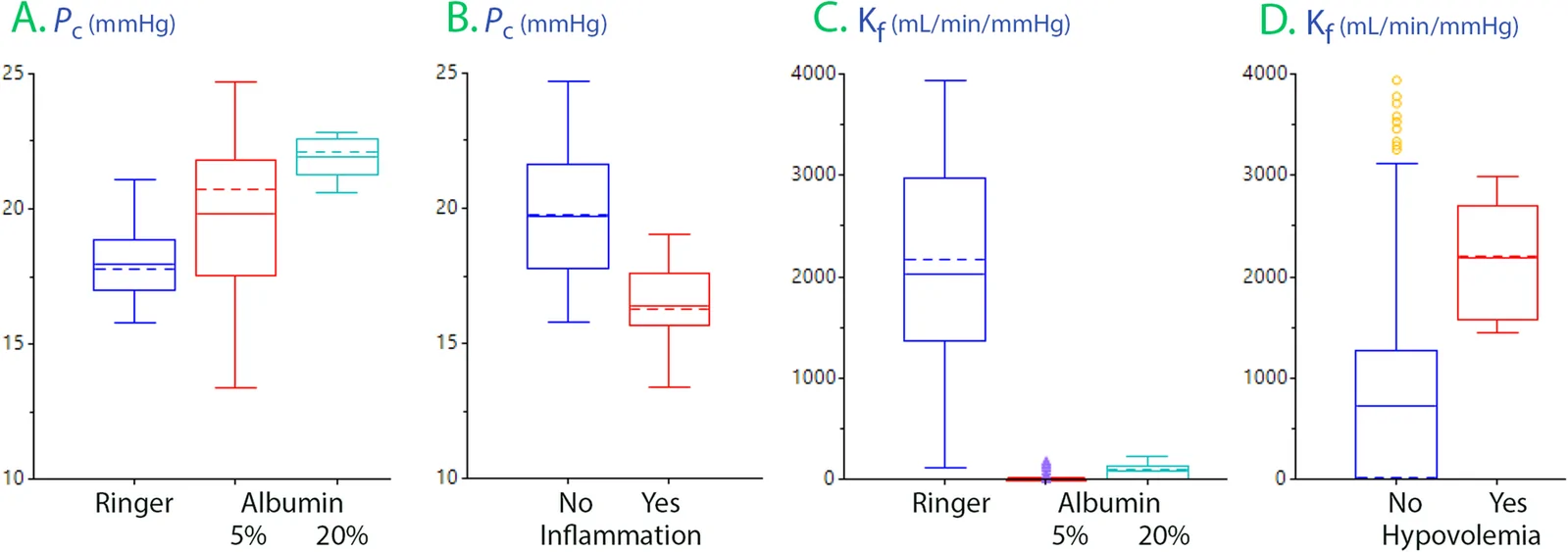
Distribution of kinetic parameters (**A**) Differences in P_c_ depending on fluid type (**B**) Differences in P_c_ depending on inflammation (only 20% albumin cases could be inflammatory) (**C**) Sharp reduction of K_f_ when albumin was administered (**D**) Increased in K_f_ in hypovolemia (only Ringer cases could be hypovolemic) Subplot B shows a statistically significant covariate while the others are association (“eta”) plots

Further additions of covariates to the model were made in an extended analysis. The use of albumin 5% was separated from albumin 20%, the influence of inflammation on P_c_ was explored, and the effect of hypovolemia on K_f_ was included (Fig. 2). *Post hoc* data from this analysis quantified K_f_ for albumin 5% to a median of 190 mL/min/mmHg while K_f_ for albumin 20% was only 4–5 mL/min/mmHg. These additions statistically improved the model but are not reported in full because the Phoenix program did not deliver any, or very wide, confidence intervals. However, the best estimates of the structural parameters of the extended analysis are reported in the top row of Table 3.

**Table 3:** **(Top row)** The Starling equation parameters after adding more statistically significant covariates to the kinetic model than shown in Table 1. The CV (between-subject variability) was 1.7% for P_c_, −7.4% for P_i_, 7.9% for π_i_, 22.4% for K_f_, and 35.5% for *σ. ***(Middle and bottom rows)**: Deviation in absolute value of each parameter from the group average in the top row depending on clinical setting. Based on post hoc and graphical data

	*P*_c_ (mmHg)	*P*_i_ (mmHg)	π_i_ (mmHg)	K_f_ (mL/min/mmHg)	σ (no unit)
All infusions	18.9	−2.18	1.44	222	0.908
*Deviation in the volunteers*					
Ringer, normovolemic	−0.7	+ 0.4	+ 0.1	+ 900	–0.12
Ringer, hypovolemic	–2.3	+ 0.3	+ 0.1	+ 2400	–0.05
Albumin 5%	+ 2.1	–2.2	+ 2.4	–35	–0.22
Albumin 20%	+ 4.5	–2.6	+ 3.2	–215	–0.23
*Deviation in the patients*					
Inflammation	–3.5	+ 2.0	–1.2	–27	–0.05
Surgery, Ringer	–2.3	+ 1.8	–1.2	+ 3200	–0.05
Surgery, albumin 20%	+ 0.4	–1.6	+ 1.9	−215	–0.20

### Association plots for clinical settings

The lower part of Table 3 was mostly derived by the “eta” vector in the population kinetic analysis, which the Phoenix program illustrates graphically as in Fig. 2. Exceptions from the use of “eta” were the covariances that had already been estimated in the extended analysis. “Eta: s” are univariate associations between covariates and the structural parameters in the kinetic model. Hence, the lower rows of Table 3 show approximations of how the structural parameters values in the extended kinetic analysis could change based on the “eta associations”.

This “eta” analyses suggest that volume loading in different clinical scenarios primarily modified P_c_ and K_f_. Their deviations from group values were largely reciprocal, the only exception being inflammation in which both P_c_ and K_f_ decreased.

### Differences between study groups

**Fig. 3:**
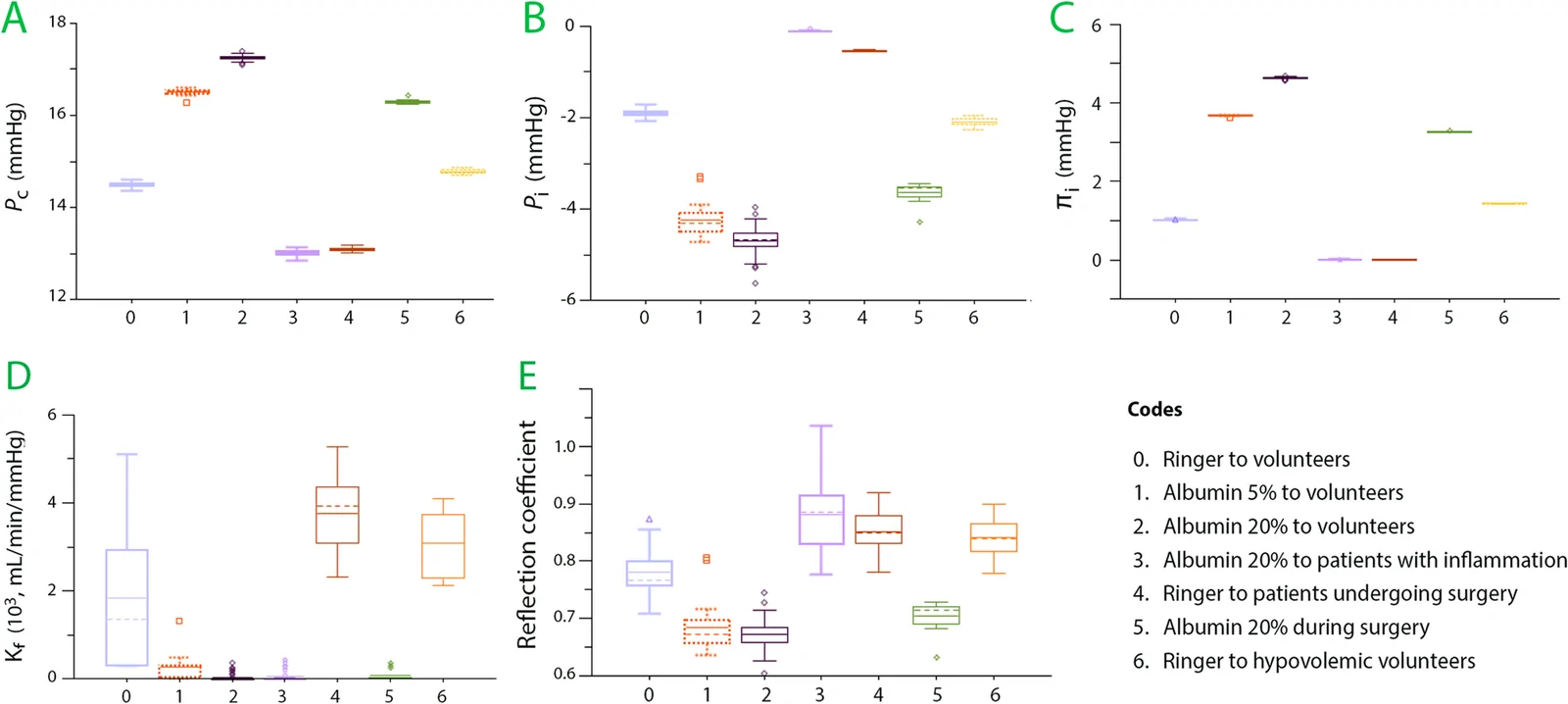
Associations (trend, or “eta plots”) suggesting variability in the parameters in the Starling equation depending on fluid type and the condition under which fluid was administered

“Eta plots” were also used to explore how the study groups differed. Technically, these plots were constructed by freezing the “best” parameter estimates obtained in Table 2 and adding a new covariate with 6 steps, each representing a study group (Fig. 3).

These plots suggested that P_c_ and π_I_ was higher in the three series of infusions where albumin was provided to non-inflammatory subjects, while these values were lower when inflammation was present (Fig. 3A, C). By contrast, P_i_ was more negative when albumin was given (Fig. 3B). K_f_ was very low in all four albumin groups (Fig. 3D). The reflection coefficient was lower when albumin was given, the exception being in the inflammatory state (Fig. 3E).

### Changes over time

**Fig. 4:**
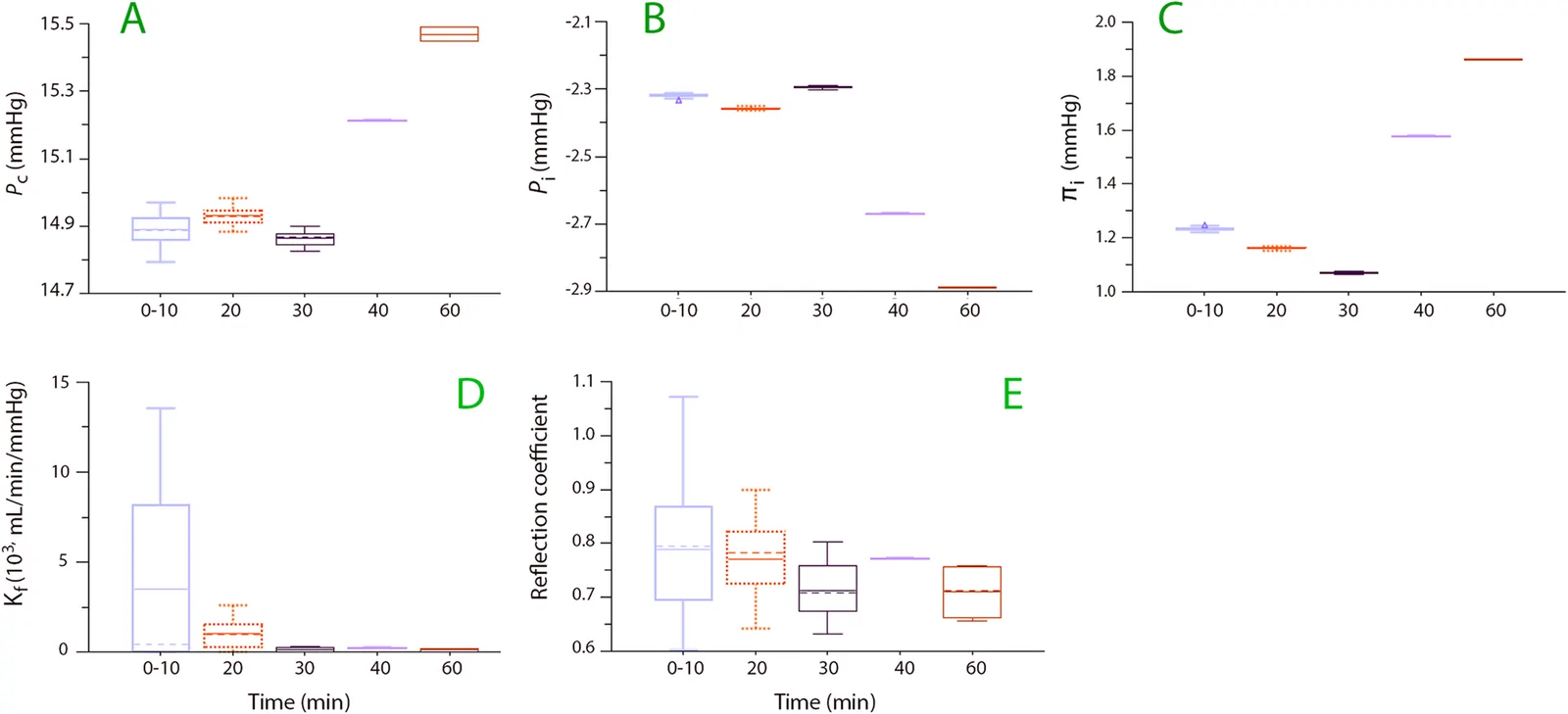
Associations (trend, or “eta plots”) suggesting variability in the parameters in the Starling equation depending on time from starting the infusion (time 0) until it ends (30 min) and the early post-infusion period (40–60 min)

Changes in the Starling parameters during the fluid infusion experiments were also studied by freezing the “best” parameter estimates obtained in Table 2 and adding a new covariate with 6 steps, but now they represented a specific period (Fig. 4). P_c_ and π_I_ was higher while P_i_ was lower post-infusion (Fig. 4A-C). Importantly, K_f_ was high in the beginning of an infusion and attained very low values post-infusion (Fig. 4D). The reflection coefficient decreased slowly over time (Fig. 4E).

### Sensitivity analysis

The estimates of the Starling parameters were perturbated 10%, 25%, and 50%. P_c_ seemed to have a second minimum at 25 mmHg, i.e. at 50% perturbation. P_i_ marginally impaired the model at 10% and 25% but a major disturbance occurred at 50%. π_I_ and *σ*, caused minor impairment of the model at a perturbation of 10% but major ones at 25% and 50%. K_f_ was least sensitive to perturbation.

## Discussion

### Key findings

The early response to rapid administration of crystalloid fluid consisted in a marked increase of K_f_ although the value decreased rapidly over 20–30 min to eventually be replaced by a modest increase of P_c_. Albumin infusion was characterized by higher P_c_ values, which is apparently needed to maintain a positive transcapillary pressure difference when π_c_ increases. Subacute inflammation was studied in the albumin 20% group and was then associated with lower K_f_ and P_c_, while P_i_ was higher than in the volunteers. Both ongoing surgery and hypovolemia were associated with a modestly high K_f,_, and surgery also with a slightly lower P_c_. The results support that π_i_ is consistently low, which agrees with the Revised Starling principle.

### The Starling parameters

The estimates of the Starling parameters agree well with data from laboratory experiments in animals.

The P_c_ of 15 to 18 mmHg does not differ much from pressures previously reported for P_c_ at the heart level, which is relevant because all subjects were studied in the supine position with blood sampled from a cubital vein. The “mean functional P_c_” has been suggested to be 17.3 mmHg [11].

Early studies by Guyton et al. found P_i_ to be slightly negative [1, 2] and typical values in the dog are − 2 to − 3 mmHg [12]. With that background, the value of − 2.2 mmHg reported here for humans is reasonable.

The colloid back-pressure (π_i_) was previously believed to equal the interstitial colloid osmotic pressure, which would be 10–12 mmHg in the present study [4, 5]. However, the Revised Starling principle suggests that it is much lower [6, 7]. Here, π_i_ was approximately 1.5 mmHg, which is only 15% of the expected interstitial colloid osmotic pressure. Inserting and freezing the higher, old value into the kinetic model and re-running the analysis yielded totally unrealistic estimates of the other parameters.

The reflection coefficient (*σ*) shows how leaky the capillaries are to plasma proteins, and the reported values of 0.8 and 0.9 are within the range of the expected 0.7 to 1.0.

Interestingly, the values of P_c_, P_i_, and π_i_ obtained in the present study are virtually identical the ones reported by Levick & Michel [6] for the cortical peritubular capillaries of the rat kidney, suggesting that the parameters in the Starling equation have not changed much during the evolution.

### The capillary filtration coefficient (K_f_)

K_f_ is the proportionality factor that aligns J_v_ with the pressure balance in the Starling equation. K_f_ was the parameter that varied most but also showed least covariance with the others. The body seems to largely regulate J_v_ by modifying K_f_ during acute volume loading. The values found here are higher than those reported elsewhere, but one must then consider that J_v_ could reach 100 mL/min at the end of the Ringer infusions, representing a 20-foldt increase from steady state, and differing greatly from the J_v_s for the albumin infusions.

K_f_ summarizes the effects of the capillary permeability for fluid (hydraulic conductivity, L_p_) and the surface area available for fluid exchange (*A*) on the transcapillary pressure balance. Increasing the albumin concentration probably decreases Lp due to its positive surface charges as well as size and volume exclusion in the capillary [13, 14]. However, the high K_f_ for crystalloid fluid is likely due to an increase of *A* from local vasodilatation induced by nitric oxide released from the glycocalyx in response to fluid-induced shear stress on the vessel walls [15]. From a phylogenetic point of view, the vascular system is not built for accommodating additional fluid at a very rapid rate, and an acute increase of K_f_ by nitric oxide would be a logical way of preventing excessive elevations of P_c_. This defense is consistent with the decrease in systemic vascular resistance that occurs in response to infusion of crystalloid fluid. On the other hand, the elevated K_f_ soon decreased, possibly due to exhaustion of the nitric oxide system. This pattern of changes in K_f_ agrees with microcirculatory findings of an increased number of perfused small vessels when plasma volume expansion is only between 1% and 5% while a reduction occurs when the volume expansion is greater [16]. However, J_v_ is always highest at the end of, and for 20–30 min after, a Ringer infusion [9], and these later adjustments must then be explained by other parameters in the Starling equation.

Ongoing surgery was associated with an unusually high K_f_, which was probably secondary to vasodilatation caused by the sympatholytic effect of general anesthesia (Fig. 3D). This allowed P_c_ to be lower during surgery than in awake volunteers. However, this was the case only when Ringer was used because K_f_ remained low when albumin was administered during surgery.

K_f_ should, logically, be reported in proportion to the body weight. The reason is that the filtration is much larger in an adult human than in a rat, while the balance between the pressure parameters in the Starling equation might be similar. Other studies in the literature do not always make a correction for body size. The basic analysis, shown in Table 2, yields a whole-body K_f_ of 4 mL/min/mmHg per kilo body weight for humans receiving an infusion of crystalloid fluid, while K_f_ for albumin was much lower.

### Physiological settings

The results include data on associations that indicate how the Starling parameters differ depending on physiological setting. Placing them into clinical scenarios and interpreting their roles is speculative but can increase our understanding of how transcapillary filtration is controlled.

The most important effect on the Starling parameters was clearly that of albumin.

The “et: s” in Table 3; Fig. 3 show that albumin infusions were associated with a low P_i_, which is probably due to oncotic-driven recruitment of interstitial fluid to the plasma via the lymph [17]. The increase in π_i_ post-infusion is probably due to wash-in of excess plasma albumin into the sub-glycocalyx space (Fig. 4C).

Other characteristics of the albumin infusions consist in high P_c_ and π_i_ while K_f_ was low. As studied here, the plasma volume expansion induced by the albumin solutions was quite similar to that obtained by Ringer´s [10]. The lower K_f_ compared to Ringer´s might then seem surprising, but the albumin infusions involved much smaller volumes. Interestingly, exchange of a lower K_f_ for higher P_c_ could be necessary here to maintain the positive transcapillary pressure gradient. Hypothetically, filtration would cease if a high K_f_ would unload P_c_ in the same way as when Ringer was administered. This adaptation might be due to a mechanistic link between K_f_ and the transcapillary pressure gradient, or else to clogging of excess albumin in the capillaries (volume exclusion) [14].

Most of the present data were recently used to quantify the restrictive effect of plasma albumin on J_v_. At similar degrees of plasma volume expansion, multiple regression models pointed at a 90% reduction of J_v_ when plasma albumin was increased by 6 g/L as opposed to diluting it by 5 g/L [10]. The estimates of the Starling parameters reported here suggest that lower K_f_ explains approximately half of this effect while changes in P_c_ and π_c_ account for the rest.

Inflammation was only assessed when albumin 20% was infused. Here, the Starling parameters differed from the typical pattern observed in non-inflammatory patients. P_c_ was low and P_i_ was high, suggesting relatively greater extravascular filling at the expense of the plasma volume. Importantly, the reduction of P_c_ shown in Table 1 represents a greater deviation than immediately apparent because P_c_ increased by 3.5 mmHg in the non-inflammatory patients receiving the same fluid. These data were derived from two sources that both represent subacute inflammation, which might explain why the reflection coefficient was unchanged. This parameter is else believed to be low in acute inflammation.

Volume loading is expected to increase P_i_ from its baseline level of approximately − 3 mmHg, and the marginal drop in P_i_ post-infusion could represent a return to baseline, as the interstitial volume decreases due to accelerated lymphatic flow at this time [8].

Hypovolemia was, understandingly, associated with a lower P_c_, but K_f_ was markedly higher in hypovolemic than in normovolemic volunteers, which was unexpected. An explanation can be that adrenergic ß_2_-stimulation due to the hypovolemic stress had already dilated the capillaries in muscle tissue when the Ringer infusion was initiated. This is a known response that promotes capillary refill in hypovolemic settings [18].

### Methodological considerations

The five unknown parameters in the Starling equation were estimated simultaneously in living humans by using kinetic analysis, which is a new approach in microcirculatory and physiological research. The results also outlined how these values are likely to change in different clinical scenarios. The output represents the “best estimate” of each parameter for the whole body throughout infusion experiments where either Ringer, albumin 5%, or albumin 20% was infused over 30 min. As mentioned previously, the analysis comprised two steps. The first one used conventional volume kinetic analysis to obtain J_v_ for each time point. The second part, which is reported here, used the obtained values of J_v_ as input data in the current mathematical analysis where π_c_ served the second known variable.

The oncotic pressure was measured in the plasma and assumed to be the same in the capillary (π_c_). Hydrostatic pressures were not measured, which is an issue because P_c_ varies throughout the vascular system and between organ to organ. However, the modeled P_c_ represents the intravascular hydrostatic pressure at the site of the fluid exchange, i.e. at the capillary level, because it is the only value of P_c_ that is in equilibrium with the other parameters in the Starling equation. Importantly, the reported value P_c_ is the average for the whole body (because J_v_ represents the whole body) albeit with greater weight given to areas where the capillary filtration is more intense. Regarding the two interstitial pressures (P_i_ and π_c_), their estimates were the two most stable parameters in this modeling work.

It might be contra-intuitive that the hydrostatic pressure on both sides of the capillary wall can be estimated by kinetic analysis based on only J_v_ and π_c_. However, this seems possible based on discrete discrepancies between the more than 3,000 estimated capillary filtration rates and measured π_c_ values and given the mathematical restraints given by the structure of Starling equation. Fluid challenges are needed that provide a wide scale of relationships between J_v_ and π_c_ for the calculations. The fact that π_c_ increases during infusions of albumin and decreases during Ringer infusions was clearly of great help to stabilize the kinetic analysis.

The estimates of the Starling parameters shown in Table 2 were derived with good confidence. However, they should not be regarded as pharmacokinetic parameters as they are not proportional to a drug concentration or, in the case of volume kinetics, to a flow of fluid. Here, there is only one flow, J_v_, and the parameters shown in the Tables rather represent a combination of values that allow fluid to pass through the capillary wall at rates represented by J_v_. A parable could be that J_v_ is like flowing water in a river where the Starling parameters have been placed out like logs at certain distances to control the flow. These distances must still be estimated using calculus as the flow constantly changes. Figure 4 suggests that the parameter values did change to some degree during an experiment, but a more apparent adaptation occurred when an infusion was turned off.

### Limitations

The output data in Tables 2 and 3 are “best estimate” values that represent all time points during a fluid balance experiment that transiently diluted the plasma by 15–25%. The values probably varied during the experiments in a way indicated by Fig. 4 but their interactions during each experiment probably created covariance, resulting in that the kinetic program could search a long time before optimal values were found. These covariances were usually positive, the exceptions being σ, for which it was negative, and K_f_, which did not show covariance.

This problem can be due to over-parameterization, but this is unlikely because the analysis was very rich in data. Both J_v_ and π_c_ during an experiment are related to the plasma volume expansion, but this is the real situation during volume loading. The limited range for the input parameter π_i_ might have contributed to the covariance (Fig. 1C). Other possible reasons include that only J_v_ and π_i_ were allowed to vary during each experiment because we don’t know how the other parameters interact. Hence, if one of the parameters changed some of the others could be expected to change, too, as they together managed the same flow. Covariance might also be evidence of plasticity of the Starling equation. This study even revealed a second set of optimal parameter values when P_c_ was 25 mmHg, but the reported set of parameters provided the highest likelihood for the model.

The study does not include baseline values because volume kinetics can only quantify flows that are induced by hemodilution. However, K_f_ would certainly attain much lower values if fluid loading had not been performed. Guyton & Hall suggests that the baseline J_v_ is probably only 2 mL/min and the transcapillary pressure gradient 0.3 mmHg, giving a K_f_ of 6.67 mL/min/mmHg [11]. By contrast, the present study yields an average pressure gradient of only 0.1 mmHg for volume loading with Ringer´s while this gradient was clearly higher for albumin 20%. However, the capillary filtration of fluid at steady state can also be inferred from the known filtration rate of albumin, which amounts to 5% of the intravascular pool per hour [19]. As argued previously [10], the interstitial colloid osmotic pressure is half as high as in plasma [5, 6] which means that the filtration of fluid occurs twice as fast, or by approximately 5 mL/min for the whole body. Nevertheless, adding this rate to J_v_ caused very small changes of the Starling parameters. Finally, the sampling site for the blood in relation to the heart might influence for the calculations. The input variable J_v_ related to the whole body but the sampled π_c_ could have been marginally influenced by gravitation.

## Conclusions

Five unknown parameters in the Starling equation were possible to quantify in living humans by kinetic analysis of the fluid distribution during volume loading. The capillary filtration coefficient (K_f_) varied most and apparently represents an important mechanism by which the body regulates the capillary filtration rate when crystalloid fluid is infused at a high rate. Albumin 5% and 20% solutions increased the capillary pressure but decreased K_f_, which suggests a clogging or a volume exclusion effect.

## Supplementary Information

Below is the link to the electronic supplementary material.

Supplementary File 1 (XLS 1.21 MB)

Supplementary File 2 (DOCX 17.4 KB)

## Data Availability

All data supporting the findings of this study are available within the paper and its Supplementary Information (Supplementary File 1.xls).
