# Supplementary material for: Estimating the Starling equation parameters in living humans by using volume kinetic analysis
Source: Pflugers Arch. 2026 Oct 12;478(11):89. doi: 10.1007/s00424-026-03219-3 (PMC13658294; doi:10.1007/s00424-026-03219-3)
Supplement: Supplementary file 1 [file 424_2026_3219_MOESM2_ESM.docx]

| Reference | Ethics committee | Infused fliuid | Database registration | Infusions  (N) |
| --- | --- | --- | --- | --- |
| Anesth Analg 2019; 129: 1232–9 | Stockholm  2014/2146-31/1 | Albumin 20% | NCT02556580 | 30 |
| Anesth Analg 2022; 134: 1270-9 | Stockholm  2014/2146-31/1 | Albumin 20% | NCT02556580 | 15 |
| Crit Care 2020; 24: 191 | Linköping  2016/333-32 | Albumin 20% | Eudra 2016-000996-26 | 15 |
| Acta Anaesth Scand 2022; 66: 847-58 Intensive Care Med Exp 2022; 10: 31 | Linköping  2017/478-31 | Albumin 5%, 20% | Eudra 2017-003687-12 | 38 |
| Clin Sci 2005; 106: 217-24 | Huddinge Hospital 115/00 | Albumin 5% | - | 15 |
| Crit Care 2013; 17: R104 | Stockholm  2010/623-32) | Ringer | NCT 01195025 | 9 |
| Anesthesiology 2005; 103: 460-9 | Huddinge Hospital 269/02 | Ringer | - | 29 |
| Anesthesiology 2002; 96: 1371-80 | Huddinge Hospital 228/98 | Ringer | - | 13 |
| Anesthesiology 1999; 90: 81-91 | Huddinge Hospital 54/95 | Ringer | - | 29 |

**Table S1.**

Literature references and ethics approvals for the studies from which data was taken to the present analysis. Complete studies were used, the only exclusions being if both plasma albumin and blood Hb values were not available.
